# Aborted Croupus Pneumonia

**Published:** 1879-11-20

**Authors:** A. G. Hobbs

**Affiliations:** Indiana


### Southern Medical Record.

EDITORS:

T. S. POWELL, M.D. W. T. GOLDSMITH, M.D. R. C. WORD, M.D,

*R. C. WORD, M.D., Managing Editor.*

☞All Communications and Letters on Business connected with the RECORD must be addressed to the Managing Editor.

VOL. IX. ATLANTA, GA., NOVEMBER 20, 1879. No. 11.

### ORIGINAL AND SELECTED ARTICLES.

### *ABORTED CROUPUS PNEUMONIA.*

BY A. G. HOBBS, M. D., INDIANA.

In your September number Dr. S. H. Anderson, of Missouri, has very kindly criticised my article on croupus pneumonia. I am glad that he did so, since the whole of his argument was upon a point that I did not dwell upon at sufficient length in my article, but he now gives me a chance to do so. The point is that pneumonitis is frequently aborted, and if so, no form of it can be due to a specific cause. The Doctor thinks it very dangerous that practitioners should be imbued with the idea that croupus pneumonia is a specific disease.

There is an old adage that "Truth hurts no man," and it applies to our science in the same, if not in a greater proportion, than to any other.

He thinks it dangerous, because in his practice he has aborted a number of cases of pneumonia. I do not doubt it in the least—I will go farther—I am confident he has often done so in his long term of practice. My experience has been much shorter than his, yet I have frequently aborted what, after accomplishing that end, I diagnosed catarrhal pneumonia. But I would ask the Doctor if he has not failed to abort many cases treated exactly in the same manner and under the same circumstances that the successfully aborted cases were treated? Has he not had even the majority of his cases to pass on through all the stages peculiar to that disease, showing all the constitutional symptoms of an ideal case of croupus pneumonia?

Perhaps in one of his aborted cases, after a careful examination, he found several lobes or even a part of both lungs beginning to be involved, while in a case where his abortive treatment failed there may have been during the whole course of the disease only one lobe or even a part of a lobe involved, and yet followed by intense constitutional symptoms. Why should his veratrum viride abort a case where more tissue is involved and fail where there is less, if the constitutional symptoms are sequent to the local inflammation in the lungs? In the one case it was the catarrhal form, and in the other the croupal.

I would ask, also, if in thinking back over his long course of practice if the Doctor does not remember that during some fall or winter when he has had a great number of cases of pneumonia in one locality (in reality an epidemic) if his abortive treatment did not seem to fail more frequently than where he had only a case occasionally?

It is difficult and even impossible to differentiate the catarrhal from the croupal variety in their incipiency. The after course of the disease—the failure to abort—the appearance of other cases in the neighborhood, proves a case of croupus pneumonia. Practitioners in malarious districts have time and again reported cases presenting all the symptoms of yellow fever, and yet no one believed them to be yellow fever, because quiuine cured or antidoted them. Since we cannot tell in the beginning whether a case will be a pneumonia due to some specific element centralizing itself in the lungs, or to a simple inflammation brought about perhaps by an irritation, a cold, or some other local cause, we should always begin with the vigorous treatment to prevent, if possible, its further extent, because we know empirically that we can often do this in purely local inflammations. If it should be the one or the other no harm is done, because if we knew in the beginning that the case was one due to a specific cause, our first object would be to control the fever if possible, because when the fever is subdued the brunt of the attack is over. Now veratrum, by its controlling action upon the heart, acts as a febrifuge, so we have not missed the mark though we have not aborted the disease.

Where, then, is the great danger that the Doctor fears, even though every practitioner in the land were imbued with the idea that croupus pneumonia were a specific disease? I do not think that because the Doctor has aborted many cases of one form of pneumonia that there cannot be another form that is specific when there are so many reasons for believing that there is such a form, which reasons I shall not repeat here, but refer him again to my article in the June number of this journal, where I endeavored to give some reasons for believing it a specific and infectious disease, none of which were answered.

